# En Coup de Sabre in a Pediatric Patient Treated With Calcipotriene

**DOI:** 10.7759/cureus.41459

**Published:** 2023-07-06

**Authors:** Alexis Coican, Derrek M Giansiracusa, Melinda F Greenfield

**Affiliations:** 1 Medicine, HCA Florida Orange Park Hospital, Orange Park, USA; 2 Medicine, HCA Florida Trinity Hospital, Trinity, USA; 3 Dermatology, Advanced Dermatology and Cosmetic Surgery - Ponte Vedra, Ponte Vedra Beach, USA

**Keywords:** systemic sclerosis, cicatricial alopecia, localized scleroderma, linear scleroderma, morphea, en coupe de sabre

## Abstract

En coup de sabre (ECDS) is a form of linear scleroderma or morphea that distinctly appears on the forehead and/or frontoparietal scalp. We report a case of a 6-year-old female that presented with a linear, hyperpigmented scar on her left forehead extending to her scalp with resultant alopecia and discoloration in the affected area. The patient was subsequently treated with topical calcipotriene ointment and had an excellent response with normalization of the sclerotic skin, hair regrowth, and improved hyperpigmentation. This report demonstrates that a conservative approach to treating pediatric patients with ECDS via calcipotriene ointment can be safe and effective.

## Introduction

Scleroderma is a condition characterized by excess collagen deposition, causing progressive fibrosis and thickening of the skin and other connective tissues [1]. Systemic sclerosis results in systemic involvement of internal organs, while local scleroderma, or morphea, involves a confined skin area. ECDS is a form of linear scleroderma that translates to "strike of the sword" and is the most common subtype in children [1]. ECDS distinctly appears on the forehead and/or frontoparietal scalp [1]. This subtype of linear scleroderma is often associated with extracutaneous neurological findings such as headaches, seizures, and visual abnormalities [2]. The incidence of linear scleroderma ranges from 0.4 to 2.7 cases per 100,000 individuals and predominantly affects women and children [1,2]. To our knowledge, calcipotriene monotherapy to treat ECDS in a pediatric patient has not been previously reported. However, a single case report utilizing calcipotriol ointment plus cream psoralen and UVA therapy in two patients with ECDS showed significant improvement in the sclerotic lesions [3]. Here we evaluate the safety and efficacy of topical calcipotriene in a pediatric patient with ECDS and associated alopecia.

## Case presentation

A 6-year-old African American female presented with a scar on her left forehead, extending to her scalp. The same area had associated hair loss and darkening of the skin. The patient denied pruritus, pain, trauma, loss of sensation, or bleeding in the area. She denied headaches, seizures, or weakness in any part of her body. The scar initially appeared as a hyperpigmented streak on the forehead, which became indurated and scar-like over six months. Since then, the scar has not changed in size, shape, or color. The patient has no significant medical history, denied recent illness, and is not currently using any prescription medication. There is no family history of scleroderma or other autoimmune diseases.

**Figure 1:**
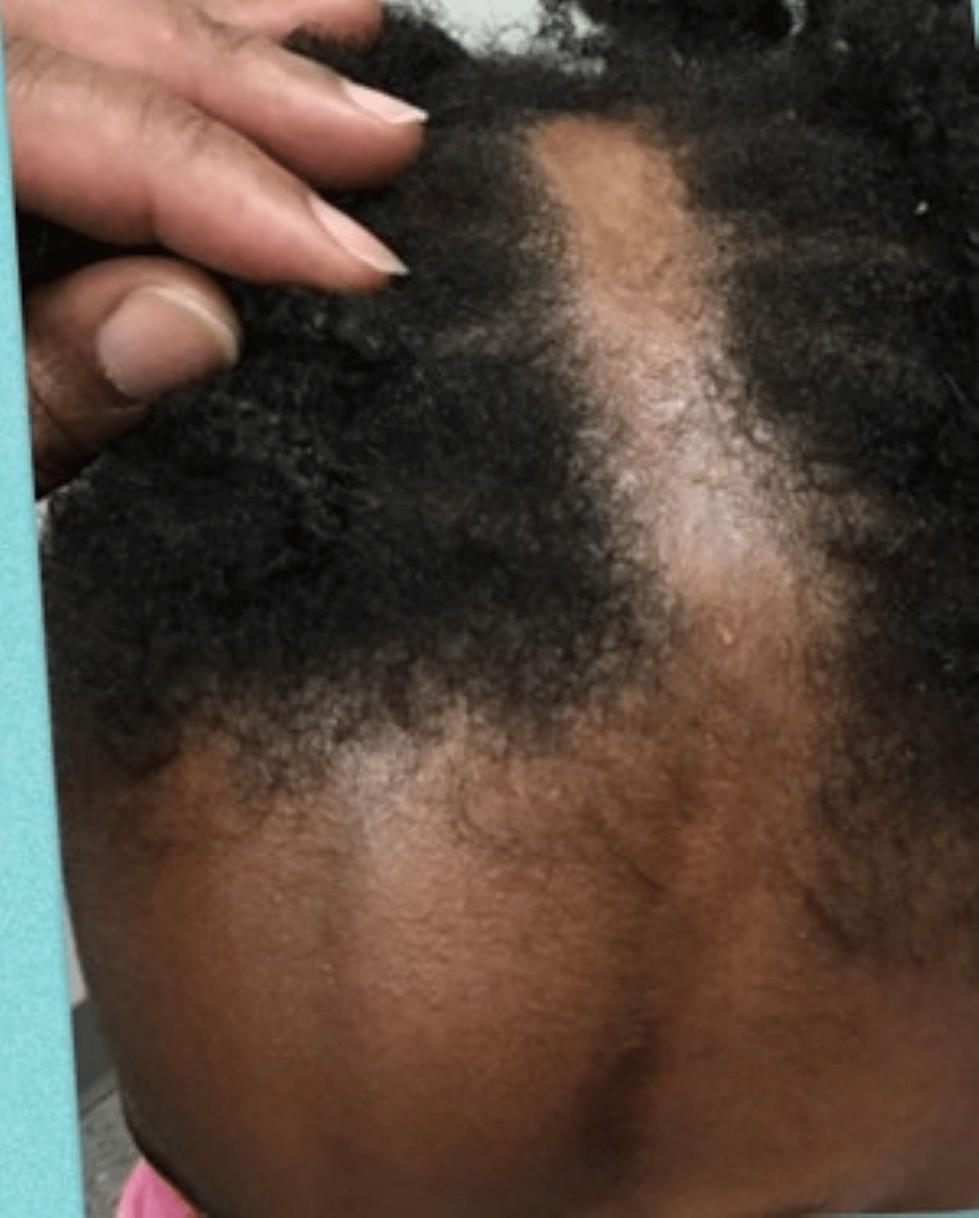
En coup de sabre pre-treatment with calcipotriene ointment Demonstrates a 10 x 2cm solitary, linear, alopecic, darkened plaque on the patient’s left forehead and left frontoparietal scalp.

**Figure 2:**
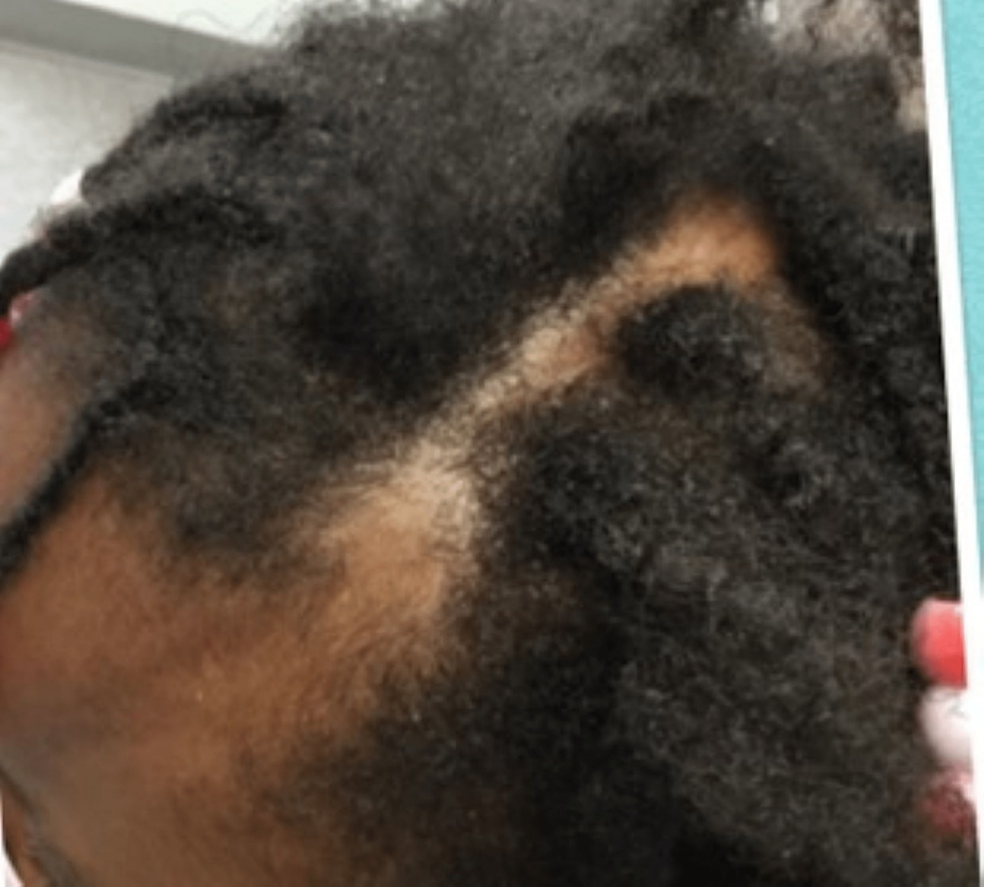
En coup de sabre three months post-treatment with calcipotriene ointment. Demonstrates improved hyperpigmentation and induration with resolving alopecia on the left forehead and left frontoparietal scalp.

Physical examination revealed an otherwise healthy young girl with a 10 x 2 cm linear, alopecic, hyperpigmented scar extending from her left forehead to her frontoparietal scalp with no facial hemiatrophy (Figure 1). The patient's mother denied biopsy at this time. A clinical diagnosis of linear scleroderma (en coup de sabre subtype) was made due to the patient's age, location of the lesion, and clinical characteristics. The patient's mother said she would like to avoid using steroids or systemic agents and asked if any other topical medications could be effective. It was reasoned that a topically applied vitamin D analog could be a safe and effective therapy for localized disease. The patient's mother was instructed to apply topical calcipotriene 0.005% ointment in the morning and the evening to the affected areas. At approximately three months of follow-up, a remarkable lesion improvement was noted, with resolving alopecia, hyperpigmentation, and softening of the affected skin (Figure 2).

## Discussion

ECDS is a rare subtype of localized scleroderma that involves the scalp and forehead. It presents as a linear, fibrous scar and may have associated pigmentary changes, telangiectasias, and alopecia [1.2]. The differential diagnoses of ECDS include focal dermal hypoplasia, birth trauma, localized morphea, lupus profundus, and progressive hemifacial atrophy or Parry Romberg Syndrome [1,2]. 

Although the exact etiology is not fully elucidated, the literature suggests localized scleroderma is a systemic autoimmune disorder with a strong correlation to a family history of autoimmune disease [4]. Several lines of evidence support CD4+ T-Helper cells, and the resultant pro-fibrotic cytokines, including TGF-β, may be involved in the progression of fibrotic changes characteristic of these lesions [5]. The histopathology of ECDS depends on the stage of the disease and the depth of involvement. Generally, more inflammatory infiltrates are seen in earlier lesions, while broad sclerotic bundles and increased dermal thickness are seen later in the disease course [6]. If the scalp is involved, cicatricial alopecia is seen with eccrine gland atrophy and perineural lymphocytic infiltration [7].

Various treatments are available for ECDS, with methotrexate and corticosteroids being the most investigated and efficacious therapy in children and adults [2,8]. The current treatment of choice for active lesions includes topical and intralesional corticosteroids. Methotrexate is useful in treating acute and deep forms of linear scleroderma and is sometimes used with steroids [2]. Other therapies include lasers, UVA1, mycophenolate mofetil, and vitamin D analogs like calcipotriene [2,8]. Calcipotriene inhibits fibroblast proliferation and collagen synthesis and downregulates T-cell function and downstream cytokine-induced fibrosis [9]. 

In a 3-month open-label study of the use of topical calcipotriene for localized scleroderma and linear scleroderma, all patients showed statistically significant improvement in hyperpigmentation, induration, erythema, and telangiectasia [10]. Furthermore, there were no adverse effects associated with treatment.

In our case above, we presented a case of a 6-year-old female presenting with a linear alopecic scar on the left frontoparietal scalp and forehead without associated hemiatrophy or neurological findings. Although the patient did not present with neurological findings, an MRI is warranted to rule out intracranial anomalies [11]. Due to the mother's reservations about using chronic steroids or systemic agents, we initiated calcipotriene ointment. Calcipotriene ointment was safe and effective in treating her lesions, which did not warrant further use of chronic steroids or systemic therapies in this pediatric patient.

## Conclusions

ECDS is a rare subtype of linear scleroderma that involves the scalp and forehead. It manifests as a linear fibrotic plaque with associated alopecia that affects the forehead and frontoparietal scalp. These lesions may be associated with ipsilateral face atrophy and neurological sequelae. Signs of neurological involvement should prompt an urgent evaluation with a brain MRI. We do not suggest that topical calcipotriene can replace steroids or systemic therapies to treat morphea or ECDS. This case exemplifies that calcipotriene can be efficacious in treating ECDS and should be considered in select patients without associated hemiatrophy or neurological findings. Further genetic and immunological studies are warranted to elucidate the underlying pathogenesis of ECDS. With the paucity of studies demonstrating effective treatment options for ECDS, clinicians are urged to share their experiences and results when treating these patients.
